# My ‘brush’ with kuru research

**DOI:** 10.1098/rstb.2008.4036

**Published:** 2008-11-27

**Authors:** Gabriele M. Zu Rhein

**Affiliations:** Department of Pathology and Laboratory Medicine, University of WisconsinMadison, WI 53706, USA

In November 1966, I received, to my surprise, formalin-fixed pieces of brain tissue from a young kuru patient (Tabaso, female, 12 years) from Dr Michael Alpers at the NIH with the request for electron microscopy (EM). At that time, I was engaged in EM studies of infectious diseases of the central nervous system, especially of progressive multifocal leukoencephalopathy (PML), a conventional slow virus disease. I was delighted by the new challenge since my interest in kuru went back already to 1960. Through an epidemiology/neurology ‘network’ starting with Dr Leonard Kurland at the NIH, and extending to Prof. Francis M. Forster, in Madison, Wisconsin I had heard of this exciting new brain disease studied by Dr D. C. Gajdusek in Papua New Guinea. I received several reprints, which I also used for my teaching, but I was not able to obtain histologic slides for review since they had been sent to the Bunge Institute in Antwerp (letter by Marion Poms, NIH, to Prof. Forster, 18 January 1960).

In the autumn of 1966, I greatly enjoyed hearing follow-ups on the kuru ‘story’ while participating in the ‘Workshop on Slow Viruses’, sponsored by the Multiple Sclerosis Society, and held at the USPHS Rocky Mountain Laboratory, in Hamilton, Montana, 12–13 September. Dr Gajdusek reported on the disappearance of kuru as a disease of childhood, and that there had been an overall 40% decrease in cases, and Dr Clarence J. Gibbs discussed the transmission of kuru to chimpanzees. I had been asked to present additional data on PML. I also brought to the group's attention the recent demonstration of paramyxovirus-like particles in subacute sclerosing panencephalitis by the French neuropathologist M. Bouteille and collaborators. The electron microscope had led again to success in the ‘slow virus’ field.

The chief aims for my kuru EM studies were a ‘hunt’ for virus particles in this transmissible disease and the description of the ultrastructure of the plaques in the cerebellar cortex. The samples of cerebellum and putamen were post-fixed in 2% glutaraldehyde and 2% osmium tetroxide. Ultra-thin sections were prepared from seven Epon blocks and were contrasted with lead citrate and uranyl acetate. They were viewed in an EMU-3G electron microscope with a highest original magnification of ×33 500.

A chance for presenting my preliminary findings came a few months later, during a symposium entitled ‘Pathogenesis and etiology of demyelinating diseases’, which took place in Locarno, Switzerland. Mrs Edith Pette, one of its organizers, had invited me to give a paper on my EM studies of PML. Several kuru investigators (Dr E. Beck, Dr E. J. Field & Dr C. J. Gibbs) had also been invited, and I got permission to speak during a discussion period (Zu Rhein 1969). Dr Beck had already summarized her light microscopic findings.

My analysis of 189 electron micrographs revealed no virus particles in either nuclei or cytoplasm of neurons or glial cells. At lower magnifications, rounded accumulations of electron-dense materials (figure 1) could be identified in considerable numbers between the granule cells of the cerebellum. Higher resolution revealed a woven pattern of these plaques (figure 2). Centrally, the meshwork of fibrils was dense and seemingly criss-crossing. However, towards the periphery, the fibrils were drawn out into radiating pointed bundles, giving the deposits a star-shaped appearance. The width of the fibrils was approximately 10 nm and when cut across (figure 2, inset), they appeared rounded or angular, and markedly electron opaque. Owing to the optical limitations of the scope, the surface details of the fibrils could not be analysed. A helical pattern seemed possible. The kuru plaques did resemble in their ultrastructure the stellate forms of amyloid as seen in murine spleens in experimental amyloidosis (Gueft & Ghidoni 1963). Owing to post-mortem changes in the kuru tissues, the relationship of the peripheral portions of the plaques to the surrounding tissues could not be established and a potential site for amyloid formation could not be suggested. In sections of the putamen, no plaques were seen. The main abnormalities were intraneuronal vacuoles containing curled membrane fragments. These vacuoles reached the size of neuronal nuclei, onto which they were sometimes juxtaposed.

Another, much more extensive study of human kuru tissue was conducted soon after mine by Field *et al*. (1969). The diameter of the fibrils was smaller, and they appeared to be hollow; and there were lattice structures in some plaques which I had never encountered. However, the authors also used a comparison with amyloid.

Subsequent to my initial work, no further collaboration was initiated by the kuru investigators. I turned my attention to transmissible mink encephalopathy, another ‘unconventional’ slow virus disease, much at home in the State of Wisconsin. Mostly together with graduate student Bob Eckroade, transmission studies to wildlife animals and to rhesus monkeys were carried out, and the clinical course and pathological features were studied (Zu Rhein *et al*. 1974). No amyloid plaques were encountered.

## Figures and Tables

**Figure 1:**
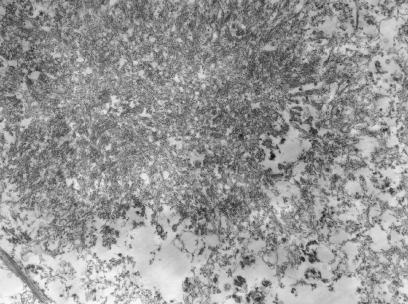
Major portion of a kuru plaque of the cerebellar cortex at a magnification of ×13 700.

**Figure 2:**
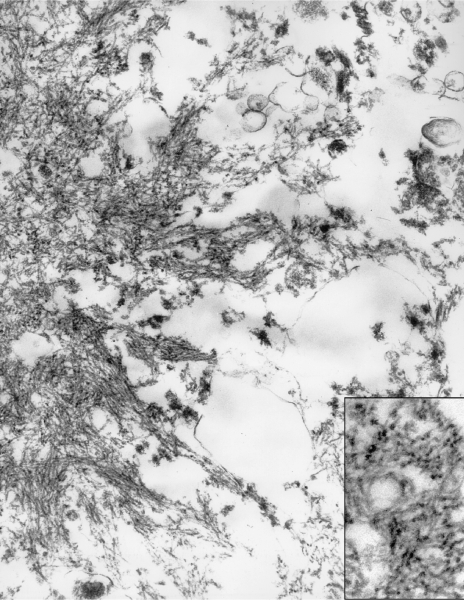
Higher magnification (×48 200) of the peripheral part of a kuru plaque revealing its fibrillar composition. Inset: area with some cross-sectioned fibre bundles ×92 000.
